# Supplementary material for: A Comparative Study of Periodontal Health Status between International and Domestic University Students in Japan
Source: Int J Environ Res Public Health. 2023 Feb 22;20(5):3866. doi: 10.3390/ijerph20053866 (PMC10001467; doi:10.3390/ijerph20053866)
Supplement: Supplementary file 1 [file ijerph-20-03866-s001.zip › ijerph-2202886-supplementary.pdf]

Table S1

	All			Domestic students			International students		
	All	Male	Female	All	Male	Female	All	Male	Female
n	231	154	77	152	118	34	79	36	43
BOP (%)	39.39	38.96	40.26	34.21	38.14	20.59	49.37	41.67	55.81
CGS	1.51	1.55	1.45	1.43	1.46	1.32	1.68	1.83	1.56
PPD (mm)	1.68	1.67	1.70	1.64	1.66	1.55	1.77	1.70	1.82

Table S2

	All		Domestic students		International students	
BOP	(+)	(−)	(+)	(−)	(+)	(−)
PPD (mm)	1.98	1.49	1.93	1.48	2.05	1.49
